# Deglutitio Impedita

**Published:** 1890-10-04

**Authors:** 


### SURGERY.

### DEGLUTITIO IMPEDITA.

Some time ago in "The Retrospect" (March 22nd) we referred to a lecture by Dr. J. Mitchell Bruce, physician and lecturer on therapeutics at the Charing Cross Hospital, on "Difficult Diagnosis." Dr. Bruce considers no less than eleven causes of difficult diagnosis, and he concludes by observing that we should approach every clinical problem in the spirit and after the method of the naturalist, not with preconceived notions, nor fettered by the artificial classification of text books, not prepared to stop when we have discovered a few symptoms or signs, or when we have found one lesion "enough to account for the symptoms, as the saying goes." In accordance with the above, and proceeding somewhat on the lines of Dr. Byrom Bramwell's "Studies of Clinical Medicine," we present the following case:—

A male patient, æt. 54, appears, complaining of difficulty of swallowing. He states it commenced rather suddenly some months back, since which time he has been unable to swallow solids, and can only swallow fluids with difficulty, and in small quantities. He does not complain of pain anywhere, considers himself otherwise in good health, but confesses to have lost flesh latterly. When asked where the food appears to stop, he points to just above the pomum Adami. There is no sense of construction round the chest, and there is no expulsion from the mouth of a morsel of food after it has passed a certain distance down the œsophagus. When asked to swallow it is apparent that he does so with difficulty. The first stage of deglutition, which is a voluntary effort, accomplished by means of the tongue and the muscles of the cheek and mouth, which takes the food as far back as the anterior arch of the fauces, seoms to be performed without difficulty. But the second stage, which is an involuntary act, although certain voluntary muscles are engaged in it, is not performed satisfactorily. The second act consists in the retraction of the tongue, the rise of the larynx, and close of the glottis, the lifting of the soft palate, the contraction of the fauces, the closure of the posterior nares, and contraction of the pharynx. It is not possible to say which of these complex movements are most faulty. All that can be said is that there is a hesitation and a prolongation of the second act of swallowing.

When this is performed, and the food passes into the œsophagus, there is no further difficulty. But when the second act of deglutition is being performed there is a slight crackling noise, evident to both patient and bystander. The speech is perfect, all the organs are healthy, the urine contains a slight amount of phosphates. There are no other symptoms.

Of course the question arising is, on what does this dysphagia, or *deglutitio impedita* depend? Careful examination of the throat shows nothing whatever abnormal. There is no redness, congestion, nor other discernible alteration internally, and there is no swelling nor tenderness externally. The patient has not suffered from an affection of the throat, such as diphtheria, tonsillitis, laryngitis, nor from mumps, nor, in fact, from any ailment for years, although recollecting a long time ago some affection of the ears, from which he quite recovered with perfect hearing. A specialist having examined the parts declares them to be healthy. Affections of the salivary glands may interfere with deglutition, and we have known difficulty of swallowing arise apparently from a so-called salivary calculus. But in this case, there is not nor ever has been, anything of the kind. Similarly the general paralysis of the insane, progressive muscular atrophy, locomotor ataxia may be connected with difficulty of deglutition, but there are no reasons whatever to suspect such conditions are even initiated. A paralysis of the glosso-pharyngeal nerve has been written about. But very little is known of the effects of paralysis of the glosso- pharyngeal nerve, for it is very rarely, if ever, paralysed alone. Moreover the nerve being the nerve of taste, the latter sense would be interferred with to a very considerable extent. In what is known as labio-glosso-pharyngeal paralysis, it has been observed that the sensory part of the nerve may escape; but then there are peculiar symptoms, the affected parts being not only the throat, but also the lips, tongue, and larynx. An early symptom is trifling indistinctness of speech. The vowels o u are not well sounded. B and p become f. Whistling is difficult or impossible. The lower part of the face loses expression. When deglutition becomes impaired, the soft palate does not close the posterior nares, so that fluids regurgitate into the nose. Food is apt to lodge in the upper part of the pharynx, and crumbs or liquid get into the larynx. Œsophageal disorders, whether functional or organic obstruction, are important causes of dysphagia. An acute œsophageal affection is out of the question in this case, so is ulceration, as there is no localised pain between the scapulæ, at the top of the sternum, nor on the præcordia. Neither are the symptoms exactly those of stricture, as there is no regurgitation of food, no sense of constriction round the chest, no glairy mucus passed, no vomiting, and no pain.

But there is one very important symptom characteristic of stricture, and that is the passage of food with a creaking sound. In the case in question there was rather a crackling sound, but no resonant gurgle as described by Allbutt. The diagnosis of incipient stricture appears therefore reasonable, and the next problem presenting is the nature of the stricture. By far the most common form of œsophageal stricture is cancerous. In this case there is a family history entirely free from any malady of the kind, and although this cannot be accepted as deciding the non-cancerous nature of the malady, it is certainly a most important point against it. There is, however, such a thing as syphilitic deposit causing stricture. It appears the patient suffered from chancre many years ago, for which he believes he took mercury, but he never had secondaries of any description. But notwithstanding this, syphilis cannot be altogether excluded as a cause, although attentive examinations and cross questioning leads to the opinion that there is not nor ever has been any other specific manifestation. The passage of a bougie ought to definitely settle any doubt as to the existence of an organic structure, besides affording information as to its site, extent, and form, but there is the fact of the specialist's declaration that there was nothing discernible the matter. Is the stricture, therefore, a spasmodic condition? The sudden manner in which the symptoms first occurred would lead to such an idea. But then the symptoms have continued without intermission ever since, and the patient is neither a young female subject to hysteria, nor a hypochondriacal man. A standard author mentions a case of simulated stricture being relieved on the removal of bleeding piles, and another on a malady of the liver being cured, but in the case in question there was neither hœmorrhoids nor liver affection. It is well known that thoracic aneurism is capable of producing all the symptoms of œsophageal stricture, but careful examination of the chest failed to detect any cardiac or arterial affection. A tubercular ulcer may cause stricture symptoms, but the patient was certainly not afflicted with any tubercular malady. Neither was there any history of swallowing caustic fluids, or even of too hot drinks. Erichsen gives the following conditions which may produce dysphagia, independently of any stricture of the canal: Tumours connected with the pharynx, morbid conditions of the larynx, tumours of the neck outside the œsophagus, aneurism of the innominate, of the internal carotid, or of the aorta, intrathoracic tumours, dislocation of the sternal end of the clavicle, impaction of a foreign body in the gullet, paralysis of the pharynx or of the larynx. In the case in question careful examination failed in detecting anything of the kind. Another standard author says, "In the early stages of the affection the diagnosis is difficult," but he adds, "an attack of spasm is perhaps the first symptom that attracts notice, this spasm completely closing the canal, and causing regurgitation of food." It has already been observed that the malady commenced suddenly, but that there has been no regurgitation of food. Altogether the diagnosis of this particular case appears too uncertain for the expression of a definite opinion; the probability seems to be commencing organic stricture from syphilitic deposit; and the prognosis is not favourable.

